# Microbial mediation of complex subterranean mineral structures

**DOI:** 10.1038/srep15525

**Published:** 2015-10-29

**Authors:** Nicola Tisato, Stefano F. F. Torriani, Sylvain Monteux, Francesco Sauro, Jo De Waele, Maria Luisa Tavagna, Ilenia M. D’Angeli, Daniel Chailloux, Michel Renda, Timothy I. Eglinton, Tomaso R. R. Bontognali

**Affiliations:** 1 University of Toronto, 35 St. George street, M5S 1A4 Toronto, (CA); 2 ETH Zurich, Institute of Integrative Biology, 8092 Zurich, (CH), now at: Syngenta Crop Protection, Münchwilen AG, Werk Stein, Schaffhauserstrasse, 4332 Stein AG, (CH); 3 Université Montpellier 2, Master EcoSystèmeS, Place Eugène Bataillon, 34095 Montpellier cedex5, (FR), now at Umeå Universitet, Climate Impacts Research Centre, 98 107 Abisko (SWE); 4 Bologna University, Department of Biological, Geological and Environmental Sciences, Italian Institute of Speleology, Via Zamboni 67, 40126 Bologna (IT); 5 ETH Zurich, ERDW, Sonneggstrasse 5, 8092 Zurich (CH); 6 FFS –Fédération Française de Spéléologie, 28 rue Delandine, 69002 LYON, (FR)

## Abstract

Helictites—an enigmatic type of mineral structure occurring in some caves—differ from classical speleothems as they develop with orientations that defy gravity. While theories for helictite formation have been forwarded, their genesis remains equivocal. Here, we show that a remarkable suite of helictites occurring in Asperge Cave (France) are formed by biologically-mediated processes, rather than abiotic processes as had hitherto been proposed. Morphological and petro-physical properties are inconsistent with mineral precipitation under purely physico-chemical control. Instead, microanalysis and molecular-biological investigation reveals the presence of a prokaryotic biofilm intimately associated with the mineral structures. We propose that microbially-influenced mineralization proceeds within a gliding biofilm which serves as a nucleation site for CaCO_3_, and where chemotaxis influences the trajectory of mineral growth, determining the macroscopic morphology of the speleothems. The influence of biofilms may explain the occurrence of similar speleothems in other caves worldwide, and sheds light on novel biomineralization processes.

For centuries, caves were considered as mostly barren and inhospitable environments to life. Only recently, thanks to the advent of new techniques in molecular biology, it has been possible to demonstrate that subsurface environments are instead populated by a vast diversity of microbes that use unconventional energy sources to perform their metabolic reactions^1^,^2^. It has been proposed that some of these microorganisms may also be involved in the precipitation of speleothems (i.e., mineral deposits that form in caves)^3^. The investigation of such mineralization processes is of interest not only for identifying and understanding new types of biomineralization pathways – that may have applications in industry – but also because these biogenic speleothems can potentially be preserved in the geological record for billions of years, becoming a useful biosignature for the search for early life on Earth and on other planets^4^,^5^,^6^,^7^. It has often been hypothesized that life arose in the dark, protected from the intense UV radiation that characterized the early Earth as well as the surface of other planets^8^,^9^,^10^. From both a paleontological and geobiological prospective, caves therefore represent a very interesting environment for the study of biomineralization processes and primitive microbe-mineral interactions.

Asperge Cave, located in the region of the Montagne Noire-Hérault (France), developed in Cambrian rock following the contact between schists and carbonates^11^. A limited portion of Asperge, the “Blue Gallery”, contains a suite of ornate white and blue speleothems resulting from the complex intertwining of numerous branches and needles of CaCO_3_ (Fig. 1, S1). Due to the presence of these “Blue-Gallery-Speleothems” (BGS), Asperge cave was proposed as a UNESCO natural world heritage site^12^. However, since its discovery in 1992, the genesis of these spectacular formations has remained unexplained^1^,^2^,^13^,^14^.

The present contribution sheds light on the genesis of the BGS showing how biological factors contribute to create such spectacular morphologies.

## Results

**Figure 1:**
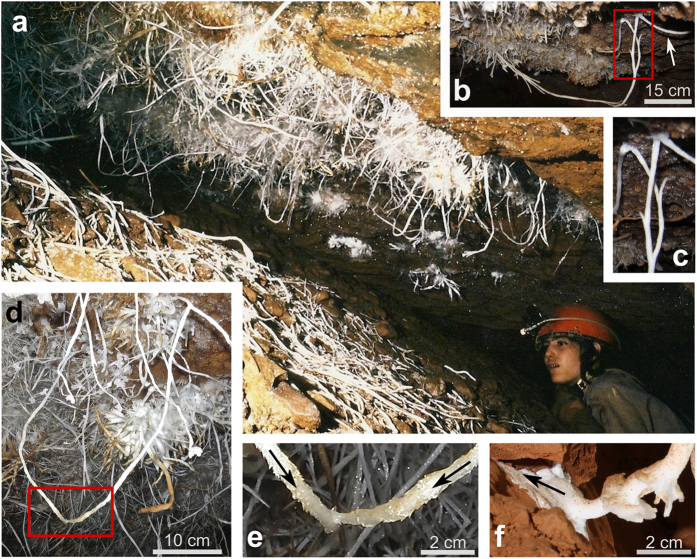
(**a**) A bouquet of Blue Gallery speleothems (BGS)—the unusual speleothems of Asperge Cave. (**b**) Astonishing “bight” connecting two points ~50 cm apart from each other. The arrow indicates a “bridge”. (**c**) BGS splitting point (detail of panel b). (**d**) Two branches meet and merge in the center of the room forming a bight (detail of panel a). (**e**) Detail of the panel d showing the “coalescence”, the arrows indicate the growing direction of the two branches. (**f**) Typical “welding point”, the arrow indicates the growth direction of the coating (i.e. against gravity). Photos by: Michel Renda, Nicola Tisato and Tomaso R. R. Bontognali.

The blue coloration of part of the BGS reflects the high Cu concentrations found in these speleothems^15^. The Cu derives from a heavy-metal-bearing geological stratum on which the speleothems grow (Fig. S2). The morphology of the BGS is very unusual and includes the following distinctive features: 1) gentle curves and “bights”, 2) bridges, 3) splitting and coalescences, 4) welding points, 5) large tubular cross-sections, and 6) preferential upward growth direction. The “bights” are composed of decimetric branches of CaCO_3_ growing downwards and orthogonally to the ceiling, then switching direction and growing upward (Fig. 1b,d). The term “bridges” refers to speleothems that span a shorter distance and are more gently curved (Fig. 1b, S1). Branching structures are evident (Fig. 1c) and, in some cases, curved speleothems, originating several decimeters apart from each other, meet and merge in the center of the gallery (Fig. 1d,e). This “coalescence” seems difficult to explain as simply coincidence. Finally, the “welding points” are sites where curbed speleothems re-join the walls of the gallery, producing a mineral coating that radiates from the contact point (Fig. 1f, S1b). Such coatings spread over the substrate without following an evident gradient, which might be imposed, for example, by gravitational or capillary flow.

**Figure 2:**
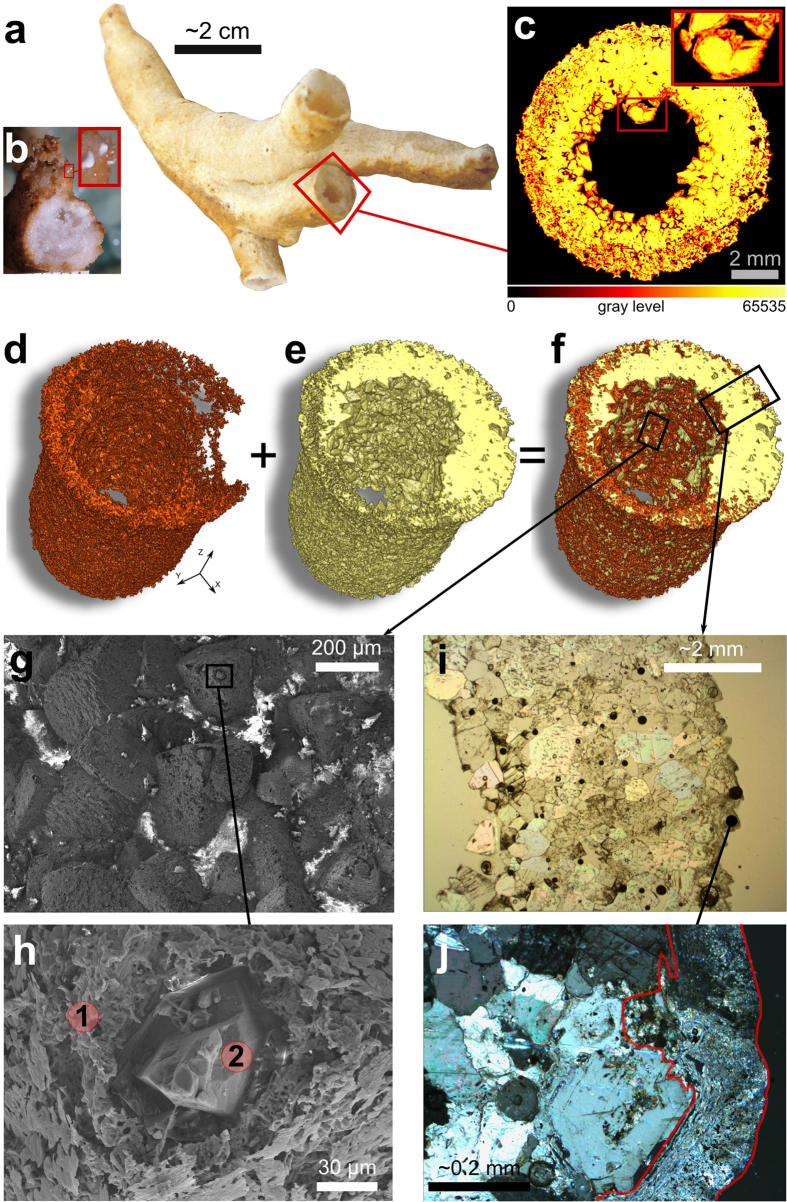
Biofilm-calcite crystals. (**a**) BGS sample having tubular morphology. (**b**) BGS sample presenting biofilms (i.e. white dots) and inner crumbly mass. (**c**) False color CT-scan image of a section of a BGS sample. Calcite crystals and biofilm are represented by pixels with gray levels ~52000 (i.e. yellow) and ~40000 (i.e. red), respectively. (**d**) The 3D model of the biofilm was obtained from the CT-scan image stack selecting voxels with gray levels between 38500 and 46500. (**e**) The 3D model of the calcite crystals was created from the CT-scan image stack segmenting voxels with gray levels between 48800 and 56800. (**f**) 3D model obtained as sum of 3D models in panel d and e. Calcite crystals are represented by the yellow solid, which is covered by the biofilm (i.e. red solid). (**g,h**) SEM images also suggest that the biofilm (detail 1 panel h) covers the calcite crystals (detail 2 panel h) (Fig. 3). (**i**) Photomicrograph of a transverse section of a BGS sample. (**j**) Photomicrograph under cross-polarized light of the external wall of BGS. Calcite crystals are surrounded by a microcrystalline mass (highlighted by the red line), which is comprised of biofilm and microcrystalline calcite (Fig. 3). Such a biofilm-calcite mixture is also suggested by the EDX analysis (Fig. 3e bottom panel). Photos by: Nicola Tisato, Francesco Sauro and Tomaso R. R. Bontognali.

**Figure 3:**
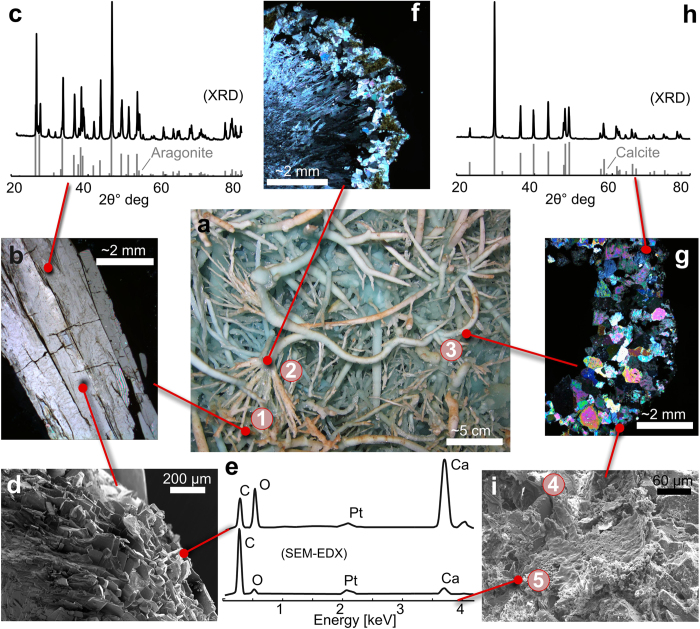
(**a**) Detail of a BGS bouquet: Hybrid speleothems (detail 2) formed between acicular speleothems (detail 1) and tubular speleothems (detail 3). (**b**) Photomicrograph under cross polarized light of the longitudinal section of an acicular speleothem, which is made of crystals growing in optical continuity (CAC). (**c**) Mineralogy of acicular speleothems was investigated with X-ray diffraction showing that these speleothems are made of aragonite. The black spectrogram is the analysis, which is compared to the aragonite XRD peaks. (**d**) SEM image of an acicular speleothem whose composition is indicated by the EDX analysis reported in panel (**e**) upper curve. (**f**) Photomicrograph under cross polarized light of the transverse section of a hybrid speleothem. The center and the rim have morphologies similar to those of acicular and tubular speleothems, respectively. (**g**) Photomicrograph under cross polarized light of the transverse section of a tubular speleothem, which is made of crystals growing in non-optical continuity. (**h**) Mineralogy of tubular speleothems was investigated by means of X-ray diffraction showing that these speleothems are made of calcite. The black spectrogram is the analysis, which is compared to the calcite XRD peaks. i) SEM image of the outer wall of a tubular speleothem which is covered by a biofilm (Fig. 2). i) EDX analysis performed on the outer wall of a tubular speleothem indicating that the biofilm is mainly made of C. However, as the EDX analysis penetrates few micrometers the surface the Ca and O peaks suggest that the first layer covering the wall is made of a carbonate mixed with organic matter (Fig. 2). Photos by: Nicola Tisato, Francesco Sauro and Tomaso R. R. Bontognali.

Shapes similar to those described above that deviate from a vertical growth axis have been previously observed at a smaller scale in other caves. They are referred to as helictites and their growth has been attributed to a combination of capillary pressure, surface energy, and gravity, requiring a central conduit with sub-millimetric diameter, and an impermeable wall^2^,^13^,^14^,^15^,^16^,^14^ (see Supplementary Information). However, most branches of the BGS have a permeable thin wall with a large inner conduit (Fig. 2) that results in a tubular morphology. Moreover, while helictite branches grow in random directions, the BGS tend to develop preferentially upward^14^. Finally, the petrography and the mineralogy of conventional helictites also differs from that of the BGS. While helictites are usually comprised of aragonite crystals grown in optical continuity (CAC) (Fig. 3b,d), the BGS are made of non-continuous calcite crystals (NCC) (Fig. 3e,g). Conventional helictites and concretions do occur in close spatial association with the BGS, often forming hybrid speleothems comprised of NCC and CAC, respectively (Fig. 3f, S1e). The coexistence of these different types of speleothems under the same physico-chemical conditions underlines the distinctive mode of mineralization by which the BGS formed.

Abiotic models that explain “non-random coalescences”, “welding points” and “tubular cross sections” in helictites do not currently exist. Moreover “U-loops”, which are structures similar to “bights”, and crumbly masses in “pool-fingers” have been previously inferred to be the result of biological factors and calcified bacterial aggregates^1^,^2^,^16^,^17^. Our investigations show that a biofilm is, indeed, associated with the BGS. Under an optical microscope such biofilms appear as a white or transparent aggregate of soft organic material. Transmitted light microscopy, scanning electron microscopy (SEM) imaging and X-Ray computed microtomography (μCT) show how the biofilm is closely associated with the crystals and almost completely cover the inner and outer wall of the BGS (Figs 2 and 3). Its presence confers to the BGS a powdery habit that differs from that of the more translucent conventional helictites present in the same cave chamber (Fig. S1). The organic composition of the biofilm was confirmed by SEM- Energy-dispersive X-ray spectroscopy (EDX) analyses (e.g. Fig. 3e).

**Figure 4:**
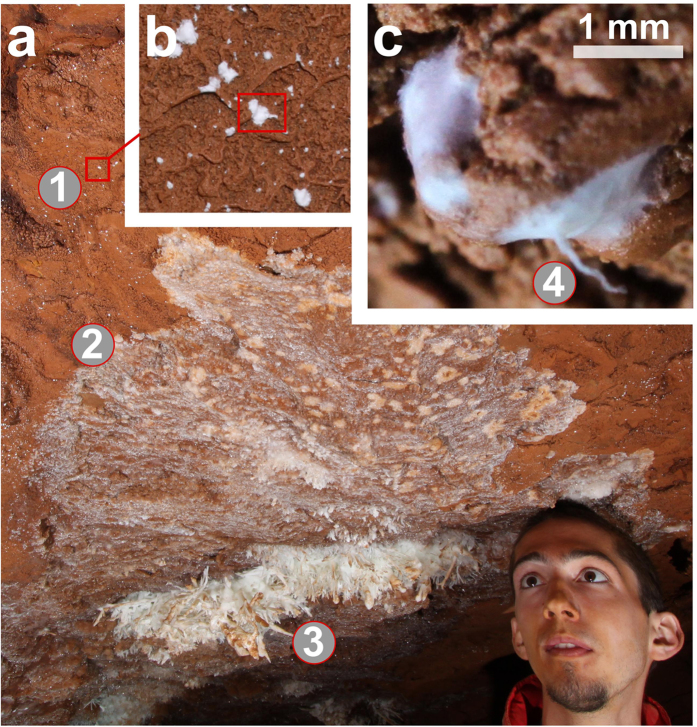
Typical sequence of features associated to the BGS. Some decimeters away from the speleothems (detail 3) aggregates of biofilm are visible as white dots (detail 1, zoom in b and c). The biofilm is locally characterized by an unusual morphology (detail 4) that may be due to the presence of microbes capable of gliding. Between the BGS (detail 3) and the mud covered by white dots (detail 1), a calcite coating covers the mud (detail 2). Aggregates of biofilm are also present below such a coating. Photos by: Nicola Tisato.

A biofilm with the same color and habit to that associated to the BGS was found also on the mud covering the walls of the “Blue Gallery”. There, thanks to the color contrast, the biofilm is visible to the naked eye as a multitude of white dots. Locally the dots develop and merge forming larger patches that show a progressive hardening (i.e., mineralization). These encrusted areas are made of CaCO_3_ and appear to constitute the initial substrate upon which the BGS develop (Fig. 4). The white dots are more abundant around BGS bouquets and exclusively occur in the Blue Gallery; only conventional speleothems adorn the other rooms of Asperge cave. These observations suggest that the biofilm may be involved in the formation of the BGS.

A diversity survey based on the 16 SrRNA gene revealed that the microbial community of the biofilm is dominated by Proteobacteria, Acidobacteria, and Actinobacteria (Fig. S3). Tests using different combinations of primers failed to reveal a significant fungal population.

Mixed cultures, inoculated *in situ* in Ca-amended agar plates directly from the BGS, induced the formation of rhombohedric dark- or light-colored crystals, recognized as calcite under optical microscopy. One of these cultures was also observed and analyzed under the SEM and EDX showing the precipitation of complex Ca-carbonate hemispheres (Fig. S4). Plates inoculated with pure cultures showed different crystals with various habits. The latter induced biomineralization of flat coating-like crystals, forming on the surface of the colonies morphologically distinct from the other previously observed using mixed cultures. These cultivation-based methods show that various bacteria that are part of the biofilm (mainly Actinobacteria) can precipitate CaCO_3_ *in vitro* (Figs S3, S4). Actinobacteria, which are often present in cave environments, have been previously documented in promoting the formation of Ca-carbonate^3^,^17^,^18^,^19^. In general, the metabolic reactions of these microbes cause a local increase in pH and alkalinity, inducing supersaturation with respect to Ca-carbonate. Moreover, the extracellular polymeric substances (EPS) that microbes produce, and that form the biofilm, act as preferential nucleation sites, influencing the morphology and the mineralogy of the precipitate^6^,^7^,^20^,^21^. Our results indicate that similar biomineralization processes may occur in the Blue Gallery. However, none of the previously described cases produce morphologically complex speleothems such as those present in Asperge, suggesting that a yet uncharacterized microorganism, or biomineralization process, may be responsible for the observed phenomenon.

## Discussion

Identifying the specific organism(s) involved may prove challenging as the majority of strains found in the BGS cannot be currently cultivated *in vitro*, and we cannot exclude that the mineralization process may be extremely slow and orchestrated by more than one species. Nevertheless, among the identified strains one is particularly intriguing as it shows 11% dissimilarity from other known 16 SrRNA sequences (i.e., potentially representing a new order of microorganism, GenBank accession number: KJ750906), and it is most closely related to Myxococcales. Some Myxobacteria species are known to efficiently induce the precipitation of calcium carbonate, as well as to tolerate high concentrations of Cu^22^,^23^,^24^. Moreover, they possess the unusual ability of gliding, a process whereby the bacterial community can move over solid substrates, forming morphologically complex swarms of cells and EPS^25^,^26^.

The presence of a biofilm, including microbes whose gliding behavior may be directed by chemotaxis, would provide a simple explanation for the distinctive morphology of the speleothems, including irregular curves, “bridges”, “bights” and upward-growing shapes. The organic material constituting the biofilm provides a nucleation site for calcium carbonate, which precipitates from the same water that the microorganisms need to survive. The factors that control the direction of biofilm growth - and subsequently the morphology of the speleothem - are difficult to be precisely identified. The biofilm may develop in random directions or in a direction that allows the microbial community to avoid being completely entombed by the carbonate mineral (e.g., preferentially upward against gravity) or, even, it may move toward the most ecologically favorable regions of the cave room (e.g., areas with higher humidity, or where localized air currents transport aerosols containing nutrient for the microorganisms). Also the “welding points” might reflect biological activity: once the microbes, located on the tip of the speleothems, reach the nutrient-rich mud, the biofilm that promotes precipitation of calcite expands following the nutrient gradient. Finally, the presence of a biofilm might also provide an explanation for the “coalescences”. Attraction between microorganisms—for instance through quorum sensing, which is typical of Myxococcales^27^—may provide a more plausible explanation than coincidental coalescence of two abiotically-formed speleothems within a large three-dimensional space.

A passive, microbially-influenced mineralization process, whereby biofilms serve as a nucleation sites for Ca-carbonate, and where biological growth—in random directions or controlled by chemotaxis—influences the orientation and trajectory of speleothem growth, represents a conservative explanation for the observed morphological features. However, we cannot exclude a more sophisticated role for the biofilm whereby microbes actively control the formation of the speleothems in order to obtain yet-to-be-identified ecological advantages. While details of the underlying processes are lacking, we conclude that the biofilm must play a key role in determining the morphology of the BGS. The occurrence of “normal” abiotic aragonitic speleothems within the same room of the Asperge cave system provides a natural “negative control”, supporting our conclusion of the biogenicity of BGS. Why the biogenic speleothem are made of calcite and not of aragonite, as well as why they occur exclusively in specific and limited areas of the cave are two aspects that deserve to be attentively evaluated in future studies. The fact that we observed hybrid acicular–tubular morphologies (Fig. 3, S1), with biofilm only associated to tubular morphologies, suggests that microbes might obtain a yet-to-be-defined benefit in converting aragonite- to calcite-speleothems. Similarly, the occurrence of the BGS exclusively in the areas of the cave that are enriched in heavy metals (e.g., Cu—commonly toxic for most living organisms) suggests that the unusual speleothems may be the result a biological detoxification process.

The results of this study may require a revised interpretation of the formation of some other types of speleothem. The remarkable similarity between the BGS and speleothems in a cave in Colorado, USA^2^ demonstrates this mineralization process is not unique. It is only in rare cases that prokaryotic microorganisms have been shown to mediate the formation of morphologically complex macroscopic mineral structures^4^. In this regard, a better understanding of the mechanisms involved may provide a basis for defining a new type of fossil structure of biogenic origin that can be recognized in the geologic record, while also yielding important insights into biomineralization processes relevant to the fields of engineering, materials science and environmental remediation^6^,^7^. Finally, as the BGS develop in an extreme environment, further investigations could be significant for studies regarding extremophiles and extraterrestrial life^5^,^8^,^9^.

## Methods

Asperge cave was visited 4 times between July 2012 and August 2013 to finalize morphological descriptions of the BGS, field observations and sampling activity. The BGS mineral structure has been extensively photographed and described in the field. Sampling of speleothems was intentionally limited to preserve the BGS bouquets.

### Light microscopy

Polished thin sections (30 μm thickness), suitable for transmitted light microscopy and SEM observation, were prepared from the original samples and analyzed with a Zeiss Axioplan Optic Microscope at the Department of Biological, Geological and Environmental Science of the University of Bologna. The microscope was equipped with a Deltapix DP200 camera for acquisition of high-resolution images.

### Sample mineralogy (XRD)

For X-Ray diffraction (XRD) analyses, few milligrams of sample were ground to an ultrafine powder and suspended in ethanol. Then a drop of suspension was deposited on a glass slide. After ethanol evaporation XRD analysis was performed using a Bruker AXS D8 Advance instrument equipped with a Lynx eye super speed detector by means of Cu K radiation, an antiscattering slit of 20 mm, and rotating sample. The sample patterns were recorded from 5.3° to 84.9° 2θ in steps of 0.033°, 2 s counting time per step.

### Scanning Electron Microscopy (SEM)

Scanning electron microscopy analyses were performed with a Zeiss Supra 50 VP at the University of Zurich, equipped with an energy-Dispersive X-ray-Detector for element analysis (EDX). A 7 nm platinum coating was applied to the samples. The image and EDX analysis were obtained with a secondary electron detector, an accelerating voltage of 15 kV, and a working distance of about 10 mm. In order to preserve the organic structures constituting the biofilm - which are usually destroyed during the drying procedure necessary to apply the metal coating—most of the analyzed samples were shock-frozen in liquid nitrogen and subsequently freeze-dried.

### X-Ray microtomography (CT-scan)

X-Ray microtomography analysis was performed using a micro-CT scanner (Phoenix X-ray V-tome-x, General Electric Sensing and Inspection Technologies) installed at the Department of Civil Engineering at the University of Toronto. The sample, a section of tubular BGS, was mounted on a 5-axis rotation stage and irradiated with X-rays on its external curved surface by rotating it 360° in 1080 equally spaced increments. At each angle, 3 projections were acquired and averaged to obtain a 2D 16-bit grey scale projection. The chosen magnification of the specimens within the field of view corresponded to a voxel resolution of ~10 μm. A 0.5 mm thick copper filter was used to reduce beam hardening artifacts in the reconstructed 3D volume.

Image reconstruction was performed using the Phoenix X-ray datos—x-reconstruction software (v. 1.5.0.22), including: a beam hardening correction of 3/10, automatic ring artefact reduction, and a ‘scan optimization’ which compensates for small translations of the specimen during scanning and correctly locates the centre of reconstruction.

Reconstruction produced an image stack formed by 1024 16-bit grey scale images with dimensions of 1018 by 1018 pixels. Segmentation of the image stack and production of the three-dimensional (3D) models were performed using the free version of the commercial software MeVisLab^28^ and includes the following passages:

1. The image stack was rescaled to a voxel size of 15 μm by means of a “lancsoz 3” filter;
2. We produced a single DICOM file from the rescaled image stack;
3. Using a “2D marked view editor”, we placed, on the DICOM file, 77 and 81 seeds (original seeds) on voxels which represented biofilm and calcite, respectively;
4. Two “region growing” procedures searched for voxels adjacent to the seeds and having grey level equal to the original seed grey level ± 6%. The new identified voxels became new seeds and the procedure was repeated until no new seeds were found. This generated two distinct volumes, representing the biofilm and the calcite, comprised of voxels with grey value ranging 38500—46500 and 48800—56800, respectively;
5. The volumes were rendered and captured as images with a “3D view” toolbox.

### Permeability of tubular BGS

The permeability of tubular BGS was qualitatively estimated using a falling head permeameter^29^. The samples consisted in two portions of speleothem ~2 cm in length. Both the samples were like small tubes with outer and inner diameter of ~1 and ~0.4 cm, respectively. Bi-component epoxy resin was applied at one of the two extremities of each tubular BGS occluding, for ~0.4 cm in length, the internal conduit. The other extremity was glued onto a 0.5 cm inner diameter silicon pipe. As a consequence, the portion of tubular BGS not sealed by epoxy resin was ~1 cm in length.

The silicon pipe was connected to the tip of a glass burette (50 ml, 0.1 ml precision). The burette was filled with ~30 ml of distilled water exerting a fluid pressure, on the inner wall of the tubular helictites, of ~40 cm of water. After <5 min water started percolating from the external wall of the helictites suggesting that the tubular helictites are rather permeable structures. However, due to the highly irregular thickness of the wall and the imprecision in defining the area of the non-sealed portion of the tubular BGS it was impossible to assess a quantitative estimation of the permeability.

### Chemical analysis with X-Ray Fluorescence Spectrometer (WD-XRF)

For wavelength dispersive X-ray fluorescence (WD-XRF) samples were ground to ultrafine powder and kept 24 hours at 110 °C. Ten grams of sample were mixed with 5 ml of solution of Elvacite polymer resin and acetone. The mixture was stirred to allow the acetone to evaporate and the resultant powder was placed in a penny-shaped mold and compressed with a vertical pressure of 40 MPa for one minute.

A WD-XRF spectrometer Axios–PANalytical (Institute for Mineralogy and Petrology - IMP of ETH–Zurich) equipped with five diffraction crystals was used for this study. The SuperQ software package provided by PANalytical was employed for calibration and data reduction. Calibration is based on 30 certified international standards. The precision of analysed elemental abundances are better than ± 0.2% for SiO_2_, ± 0.1% for the other major elements. For trace elements, relative errors are better than 10% for concentrations of 10–100 ppm, better than 5% for higher concentrations and can reach as much as 50% at levels below 10 ppm. Therefore the detection limit is considered to be approximately 5–10 ppm.

The measured sample returned total element concentrations of less than 100%. This discrepancy is related to the possible presence of water and organic matter in the sample. In addition, elements lighter than Na cannot be recognized with WD-XRF^30^.

### *In vitro* Ca-carbonate precipitation

Microorganisms associated with the BGS were sampled using a cultivation-based method. Samples of biofilm associated to BGS were scratched out directly into yeast peptone agar plates (YPA; yeast extract 3 g/L, peptone 5 g/L, agar 18 g/L) using a sterile lab spatula. Bacterial strains showing distinct phenotypic characteristics were transferred several times on yeast tryptone agar plates (YTA; yeast extract 3 g/L, tryptone 5 g/L, agar 18 g/L) to get single pure bacterial colonies.

Mixed and pure bacterial strains isolated from the BGS were tested for their ability to induce Ca-carbonate precipitation *in vitro* on YTA, amended with 3.16 g/L Ca (CH_3_COO)_2_•H_2_O. Negative controls consisting of Ca-amended YTA medium inoculated with sterile H_2_O allowed us to rule out contamination during inoculation and growth. After inoculation, plates were incubated in the dark at 19 °C for 2–3 weeks.

### 16SrRNA sequencing analysis and taxonomy of the microbiome

We characterized the microbial diversity of the white biofilm associated to the BGS via targeted 16S rRNA gene sequencing. Total DNA was extracted using the FastDNA SPIN Kit for Soil according to the manufacturer’s instructions and quantified with a NanoDrop ND-1000 spectrophotomer. Universal primers 27F (5′-AGA GTT TGA TCM TGG CTC AG-3′) and 1492R (5′-TAC GGY TAC CTT GTT ACG ACT T-3′) were used to amplify by PCR 16S rRNA. Combination of ITS5-4 and ITS1-4 primers was used to characterize the Internal Transcribed Spacer (ITS) of eukaryotic species (ITS5, 5′-TAC GGY TAC CTT GTT ACG ACT T-3′; ITS4, 5′-TCC TCC GCT TAT TGA TAT GC-3′; ITS1, 5′-CC GTA GGT GAA CCT TGC GG-3′). PCR conditions were initial denaturation at 98 °C for 30 s followed by 35 cycles including denaturation at 98 °C for 8 s, hybridization at 65 °C for 30 s and elongation at 72 °C for 25 s. A final elongation lasted 6 min at 72 °C. Phusion high fidelity DNA polymerase was used. 16S rRNA PCR and a negative control PCR sample were cloned using Clone Jet PCR kit (Thermo Scientific). DH5α *E. coli* strains were transformed by thermal shock, at 42 °C for 45 s. Transformed cells were then incubated on ice for 2 min and 950 μL of SOC medium was added. Samples were incubated at 37 °C on a horizontal shaker at 225 rpm for 1 h. Samples were inoculated on yeast-tryptone agar plates amended with ampicillin (100 μg/mL) and incubated overnight at 37 °C. Single colonies were transferred to 50 μL ddH_2_O and heat shocked for 10 min at 95 °C to burst bacterial cells and release DNA. A total of 4 μL were used to amplify the cloned insert using DreamTaq polymerase (Thermo Scientific) and primers pJET 1.2_F (5′-CGA CTC ACT ATA GGG AGA GCG GC-3′) and pJET 1.2_R (5′-AAG AAC ATC GAT TTT CCA TGG CAG-3′). Amplicons were purified through NucleoFast® PCR plates (Macherey-Nagel) and sequenced using either 27F or 1492R primers. Sequencing reactions were performed using BigDye Terminator v3.0 ready reaction cycle sequencing kit (Applied Biosystems). The sequencing PCRs were in a total volume of 10 μL using 20 to 40 ng DNA, 10 pmol of primer and 2 μL BigDye reaction mix previously 1:4 diluted. The cycling profile was 10 s denaturation at 95 °C, 5 s annealing at 50 °C and 4 min extension at 60 °C for 100 cycles. Sephadex G-50 DNA Grade F (Amersham Biosciences) was used to clean sequencing reaction prior loading the samples into a 3100 ABI automated sequencer. Sequences were quality checked and visualized using Sequencher version 4.2 software package (Gene Codes Corporation). Sequences were blasted to the NCBI database using BLASTN algorithm^31^ for similarities to previously deposited species.

No amplifications were obtained using different ITS primer combinations (ITS5-4 and ITS1-4), suggesting fungal ecology to be absent or a limited fraction of the microbiome.

16SrRNA from pure cultures cultivated *in vitro* was amplified using a high-fidelity polymerase KAPA 3G Plant PCR Kit (Kapabiosystems) and primers 27F and 1492R. PCR cleaning and sequencing was described above. Pure culture strains isolated from the BGS belonged to *Sphingopyxis* and *Rhodococcus* species (Fig. S3).

## Additional Information

**How to cite this article**: Tisato, N. *et al.* Microbial mediation of complex subterranean mineral structures. *Sci. Rep.* **5**, 15525; doi: 10.1038/srep15525 (2015).

## Supplementary Material

Supplementary Information
