# Supplementary material for: Microbial mediation of complex subterranean mineral structures
Source: Sci Rep. 2015 Oct 29;5:15525. doi: 10.1038/srep15525 (PMC4625141; doi:10.1038/srep15525)
Supplement: Supplementary Information [file srep15525-s1.pdf]

Title: Microbial mediation of complex subterranean mineral structures

Authors:

Nicola Tisato[1], Stefano Torriani[2], Sylvain Monteux[3], Francesco Sauro[4], Jo De Waele[4], Maria Luisa Tavagna[5], Ilenia M. D'Angeli[4], Daniel Chailloux[6], Michel Renda[6], Timothy I. Eglinton[5], Tomaso R. R. Bontognali[5]

Supplementary Information:

Classic helictites vs BGS:

The aim of this paragraph is to: i) review the present theories about the genesis of helictites and ii) underline the inconsistencies between these theories and the observations performed on the BGS and reported in this contribution.

Helictites (or excentric speleothems) have been documented in many cave systems around the world. They consist in twisting and turning finger-like speleothems growing in random directions, with a diameter ranging 0.1 - 10 mm and extending to some decimeters in length[2].

Helictites are believed to form from a thin capillary water film flowing through a narrow inner conduit (0.01-0.5 mm in diameter). The wall of the helictite i) is composed of acicular crystals growing in optic continuity, and ii) has a very low hydraulic permeability, allowing a relatively high hydrostatic pressure [13]. Huff suggested that specific helictite morphologies can grow as the result of abiotic carbonate deposition involving CO_2 degassing or loss of solvent (i.e. water evaporation) [13]. These processes are expected to be most prevalent at the tip of the helictite, where the water exits the conduit, or where the water film has the highest curvature and rates of CO_2 loss

and/or water evaporation [16]. Most helictites are interactive aggregates according to the ontogenic classification schemes [14].

Helictites can be mono- and polycrystalline, but are often composed of only a few crystals, made in general of aragonite or calcite. Aragonite crystals tend to undergo the process called splitting (e.g. acicular speleothems associated with the BGS), which is considered the main mechanism causing the bifurcation of helictites. In the case of two splitting crystals composing the helictite the conduit is lenticular, and its partial obstruction causes the helictite to divide in a planar way. Symmetry of the obstruction affects the feeding of the two individuals, causing abandonment of one branch when one of the conduits is too narrow to allow water to penetrate. If the helictite is composed of numerous crystals, the cross section of the feeding canal will be polygonal, and bifurcations can happen in three dimensions.

The growth direction of interactive aggregates, such as most helictites, is considered to be controlled by random effects. However crystals can undergo selection, being advantaged or disadvantaged during their growth, and therefore controlling the overall growth direction of the helictite. Competition between single growing crystals can be direct and indirect. In the first case the water film has greater mobility or supersaturation, and crystals can touch each other (interactive aggregates). In the latter case, the water film is partly isolated and changes in the local environment can directly influence the chemical properties of the water film, slowing down the crystal growth and eventually stopping it before the crystals touch each other. In most helictites, where the solution is fed continuously, competition is generally direct.

These general rules explain why most of the previously described helictites tend to grow toward the free space in the cave, and generally perpendicular to the starting point of growth. Splitting, obstruction of the feeding canal or local morphology of the growing tip can explain a random

deviation from this perpendicular growth, but not the preferential upward direction of the BGS. An explanation of “bights”, bridges, welding points and non-random “coalescences” by purely abiotic processes is also lacking. Moreover, the process described for conventional helictites growth requires an extremely low permeability of the helictite walls, allowing for maintenance of a relatively high hydrostatic pressure in the inner channel. This requirement is not respected in the BGS, whose walls are characterized by high permeability and calcite crystals devoid of optical continuity.

Figures and figure captions of the Supplementary Information:

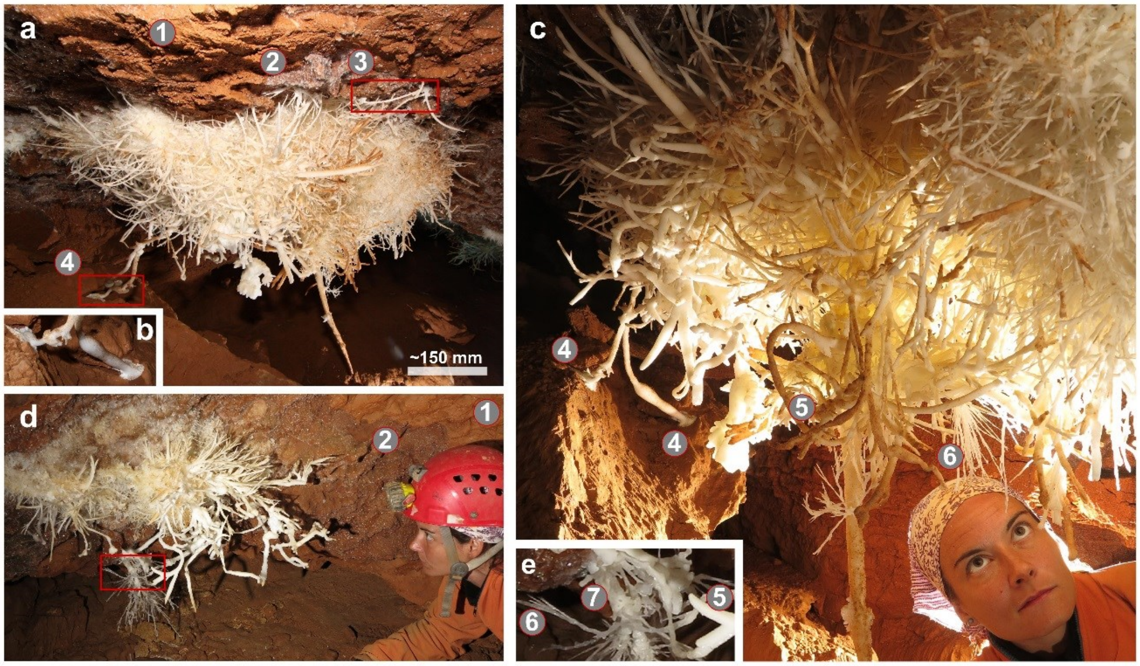

Figure S1. a) BGS bouquet. The sequence of features described in Fig. 3 can be recognized also around this bouquet: mud with white dots (detail 1), calcite coating (detail 2) and BGS (detail 3). b) Detail of "welding points" from detail 4. c) BGS bouquet, details 4, 5 and 6 are welding points, tubular and acicular morphologies, respectively. d) BGS bouquet surrounded by the typical sequence of features recognized also in Fig. 3 and panel a: mud (detail 1) and calcite coating (detail 2). e) Detail from panel d: acicular (detail 6), hybrid (detail 7) and tubular (detail 8) morphologies. Photos by: Nicola Tisato.

	%
SiO_2	38.9
CaO	24.8
Fe_2O_3	16.2
Cu	10.0
Al_2O_3	2.1
S	0.8
K_2O	0.5
Others	0.4

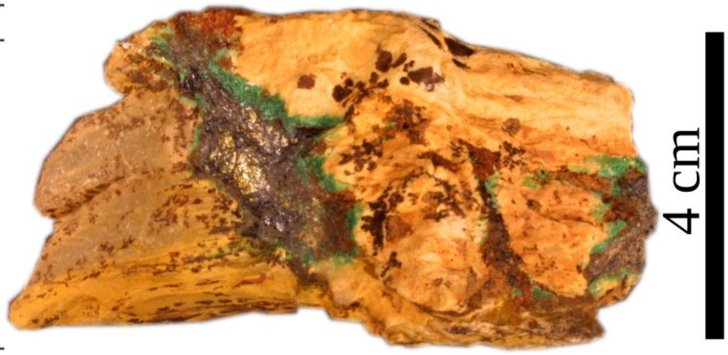

Figure S2. Sample AS14 was extracted from a layer of schist overlying the ceiling of the "Blue Gallery". The table reports the WD-XRF analysis, which reveals the high concentration of metals. Photo by: Nicola Tisato.

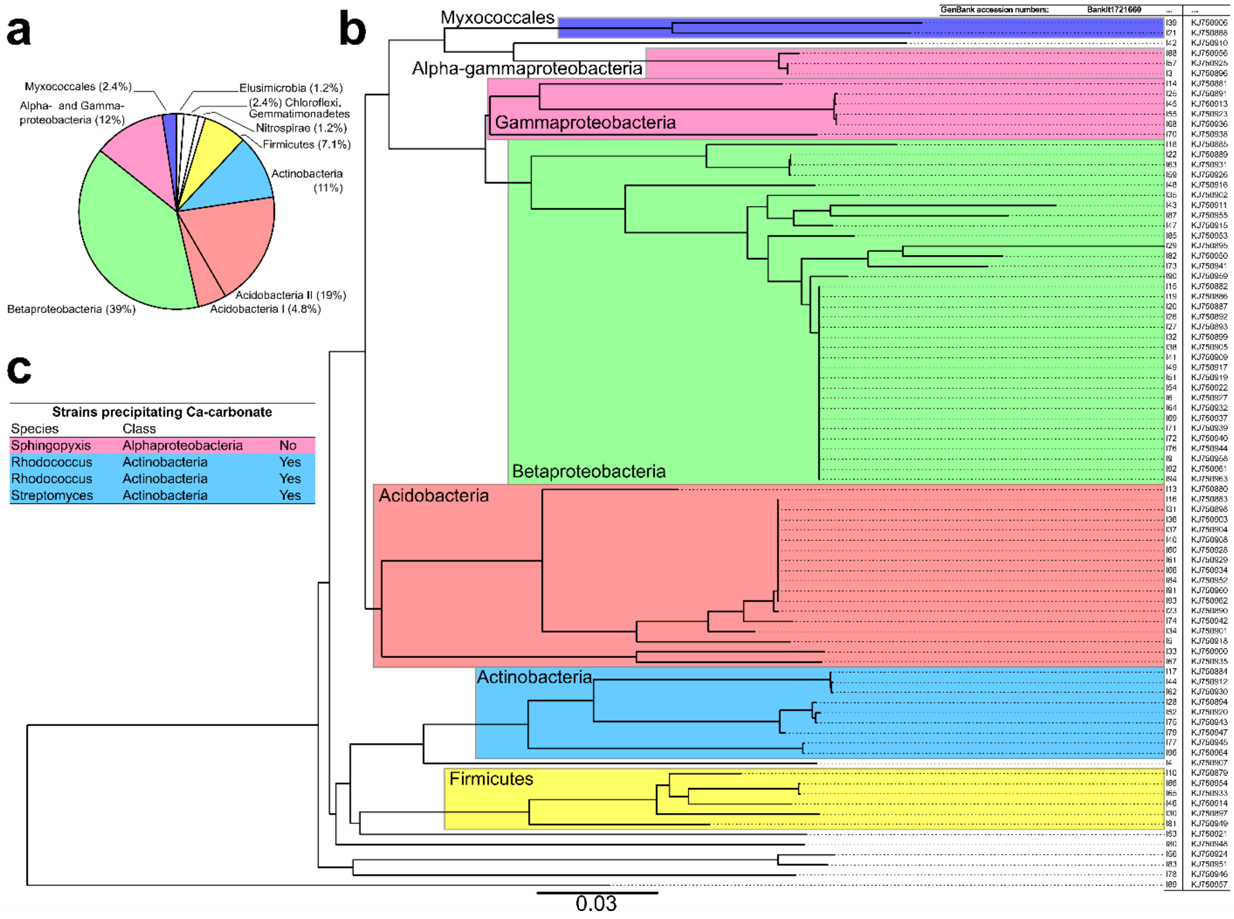

Figure S3. a) The BGS microbiome presented as pie chart showing the relative frequencies of bacterial classes and/or phyla identified by blasting the 86 analyzed 16s RNA sequences. The most frequent phylum was Proteobacteria (51%). Within Proteobacteria the class of Betaproteobacteria (green) was predominant, making 39% of the total BGS microbiome. Alpha and Gammaproteobacteria together (pink) made 12% of BGS. Acidobacteria (salmon pink), Actinobacteria (light blue) and Firmicutes (yellow) characterized 24%, 11% and 7% of BGS, respectively. a) Phylogenetic dendogram (Neighbor-Joining) inferred from the obtained 16S rRNA sequences aligned using MAFFT and the integrated Q-INS-i option. The scale bar represents 0.03 substitutions per nucleotide positions. The colors highlight the same bacterial phyla and/or classes as in (a). All sequences were submitted to NCBI following the accession numbers listed on the right of the Phylogenetic dendogram. c) Four bacterial strains isolated from BGS, belonging to three species (*Sphingopyxis*, *Rhodococcus* and *Streptomyces*) and two phyla (alphaproteobacteria and actinobacteria) were tested for their ability of precipitating Ca-carbonate *in vitro.*

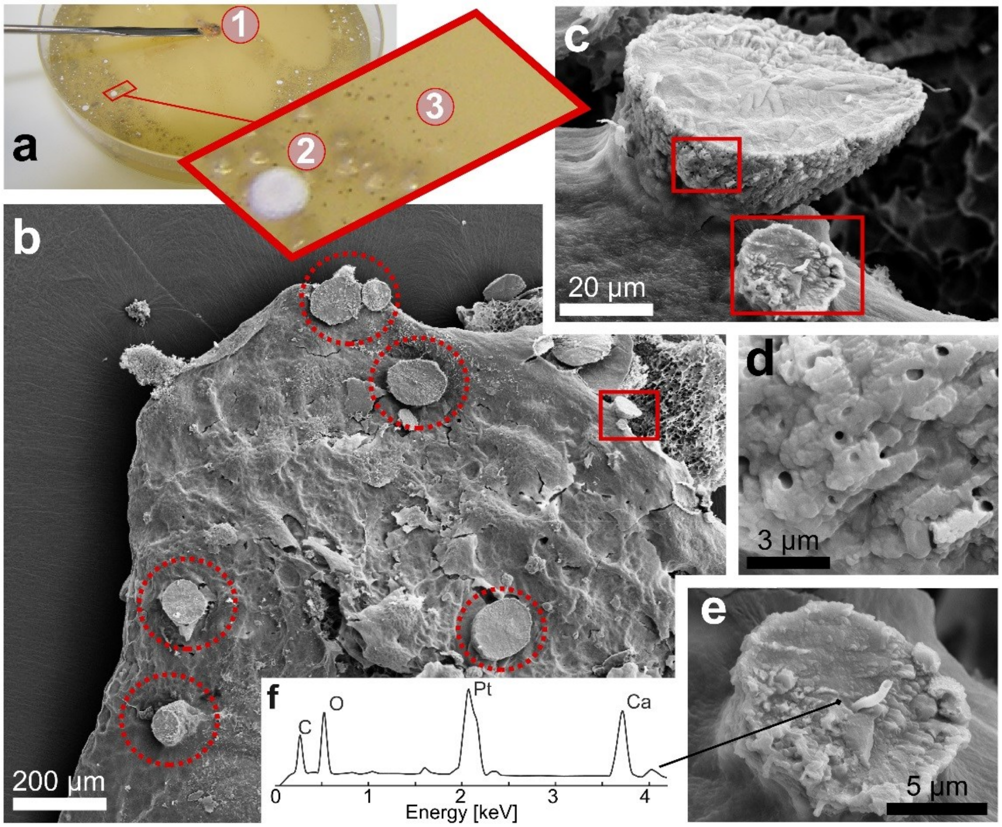

Figure S4. In vitro experiment. a) Ca-amended agar plate inoculated with a biofilm (white dot) previously isolated from the ceiling of the "Blue Gallery". The agar plate was kept at 19°C in darkness for 2 weeks. After this period white dots had grown extensively on the agar plate (detail 2) together with brown nodules (detail 3). A white dot and some brown nodules were collected from the agar and examined under SEM (panel c). The sample was placed up-side down on the sample stage. Brown dots are the hemispheres highlighted by red circles and detailed in panel c, d and e. The spongy structure visible on the upper right corner of panel c is the biofilm (i.e. white dot). Mineralogy of the hemispheres (i.e. $CaCO_3$) is confirmed by the EDX (panel f). Calcite hemispheres exhibit nanometric holes (panel d), and tubular structures (panel e) on the curved side and the base, respectively. Photos by: Nicola Tisato and Tomaso R. R. Bontognali.
